# Modulation of T-cell function by myeloid-derived suppressor cells in hematological malignancies

**DOI:** 10.3389/fcell.2023.1129343

**Published:** 2023-04-05

**Authors:** Vaishali Bhardwaj, Stephen M. Ansell

**Affiliations:** Division of Hematology and Internal Medicine, Mayo Clinic, Rochester, MN, United States

**Keywords:** myeloid-derived suppressor cells, hematological malignancies, tumor microenvironment, t cells, immunosuppression

## Abstract

Myeloid-derived suppressor cells (MDSCs) are pathologically activated neutrophils and monocytes that negatively regulate the immune response to cancer and chronic infections. Abnormal myelopoiesis and pathological activation of myeloid cells generate this heterogeneous population of myeloid-derived suppressor cells. They are characterized by their distinct transcription, phenotypic, biochemical, and functional features. In the tumor microenvironment (TME), myeloid-derived suppressor cells represent an important class of immunosuppressive cells that correlate with tumor burden, stage, and a poor prognosis. Myeloid-derived suppressor cells exert a strong immunosuppressive effect on T-cells (and a broad range of other immune cells), by blocking lymphocyte homing, increasing production of reactive oxygen and nitrogen species, promoting secretion of various cytokines, chemokines, and immune regulatory molecules, stimulation of other immunosuppressive cells, depletion of various metabolites, and upregulation of immune checkpoint molecules. Additionally, the heterogeneity of myeloid-derived suppressor cells in cancer makes their identification challenging. Overall, they serve as a major obstacle for many cancer immunotherapies and targeting them could be a favorable strategy to improve the effectiveness of immunotherapeutic interventions. However, in hematological malignancies, particularly B-cell malignancies, the clinical outcomes of targeting these myeloid-derived suppressor cells is a field that is still to be explored. This review summarizes the complex biology of myeloid-derived suppressor cells with an emphasis on the immunosuppressive pathways used by myeloid-derived suppressor cells to modulate T-cell function in hematological malignancies. In addition, we describe the challenges, therapeutic strategies, and clinical relevance of targeting myeloid-derived suppressor cells in these diseases.

## 1 Introduction

Myeloid cells are highly diverse cells that are essential for the efficient functioning of innate and adaptive immunity. These cells are divided into two categories (a) monocytic myeloid cells which include monocytes, macrophages, and dendritic cells (DC), and (b) granulocytic/polymorphonuclear (PMN) myeloid cells including neutrophils, eosinophils, basophils, and mast cells. Under steady-state conditions, hematopoiesis is a well-regulated process that includes a series of cell lineage commitments, defined steps of cell differentiation (dedicated transition of hematopoietic stem cells to lymphoid cells/myeloid cells), and lastly, maturation/circulation of immune cells. During stress/pathological conditions such as cancer or infection, certain signals derived from the hematopoietic stem cell (HSC) niche modify the fate of HSCs, known as “emergency hematopoiesis”. This emergency hematopoiesis results in increased demand for both lymphoid and myeloid cells. The persistence of emergency myelopoiesis results in the accumulation of immature myeloid cells/neutrophils/monocytes which are characterized by their immune suppressor activity, now known as myeloid-derived suppressor cells (MDSCs). The immunosuppressive activity of neutrophils and monocytes was first reported around 30 years ago and these immunosuppressive cells were later named MDSCs (Gabrilovich et al., 2007). In healthy individuals, MDSCs are present in very low numbers and are involved in regulating immune responses and tissue repair (Gabrilovich and Nagaraj, 2009). However, MDSCs rapidly expand attesting to their critical role during pathological conditions such as chronic infections, autoimmune disorders, and cancer.

Given the adverse effects of MDSCs, the question arises as to why this population is evolutionary conserved. The first report by Köstlin et al. (2017) reported the expansion of polymorphonuclear MDSCs (PMN-MDSCs) during human pregnancy and the modulation of T-cell response. This provides maternal tolerance for the allogenic fetus. During pregnancy PMN-MDSCs accumulate in the peripheral blood of the mother and the human placenta/cord blood of the newborns. However, after giving birth, the mother’s MDSCs revert to their normal levels with the polarization of T-cell association with MDSCs (Köstlin et al., 2016). Additionally, Ostrand-Rosenberg and Fenselau (2018) found that MDSCs serve as a frontline barrier (in placenta) and facilitate maternal-fetal tolerance. These studies attest to the fact that MDSC’s play an important role in pregnancy and neonates (Veglia et al., 2018; Dorhoi et al., 2020).

On the other hand, activated MDSCs participate in several aspects of angiogenesis, tumor growth and metastasis, premetastatic niche formation, and epithelial-mesenchymal transition (EMT). The tumor microenvironment (TME) plays a critical role in supporting the growth and differentiation of MDSCs. The TME is a complex network of cellular and non-cellular components around the tumor cells which consists of lymphocytes, other immune cells, stromal cells, and extracellular matrix (ECM). The TME provides a permissive environment for tumor growth, invasion, and metastasis. The composition of TME in B-cell malignancies in divided into two parts: The immune microenvironment and the non-immune microenvironment (discussed below in section 4.1). The bidirectional interaction of MDSCs and tumor cells creates a haven in TME for the tumor growth. Accumulating evidence supports a pivotal role of MDSCs in cancer progression and immune suppression. Recent studies have reported the importance of the TME and the expansion of MDSCs in the pathogenesis of B-cell lymphoma (Höpken and Rehm, 2019; Liu et al., 2021). Briefly, B-cell lymphomas are a group of hematological malignancies that are characterized by complex clinical and biological heterogeneity. Lymphomas comprises Hodgkin lymphoma (HL) and non-Hodgkin lymphoma (NHL). Further, B-cell lymphoma constitutes for almost 95% of the total NHL cases. (Ennishi et al., 2020; Sehn and Salles, 2021).

In this review, we will discuss the phenotypic characteristics of MDSC, along with their genomic and metabolic profiles. We also describe how the characteristic features of MDSCs can be potentially used for therapeutic purposes in B-cell lymphomas. However, due to the short lifespan (Condamine et al., 2014) and immune escape properties of MDSCs, the therapeutic strategies in B-cell malignancies are currently somewhat limited. Therefore, we highlight the gaps in the literature which can be explored in emerging studies that will help to limit the severity of the disease. In summary, we define the challenges and clinical relevance of targeting MDSCs in hematological malignancies.

## 2 Biology, development and differentiation of myeloid-derived suppressor cells

Hematopoietic stem cells (HSPCs) reside in bone marrow and differentiate into all cell types including lymphoid cells, myeloid cells, and red blood cells (RBCs.) Common myeloid progenitor cells in bone marrow generate myeloid-derived suppressor cells (MDSCs). The generation of MDSCs is considered as a two-phased, somewhat overlapping process,—(a) expansion of myeloid cells and (b) procurement of MDSCs features by myeloid cells. Generally, MDSCs develop in bone marrow, however, their generation may also take place in other organs including spleen or liver as demonstrated in tumor bearing mice (TB mice) and in patients diagnosed with cancer (Tavukcuoglu et al., 2020).

Classically, bone marrow derived immature myeloid cells (IMCs) differentiate into granulocytes, dendritic cells and macrophages. However, during demand adapted myelopoiesis (or emergency myelopoiesis), there is robust expansion of IMCs. If the inflammation is resolved, the mechanism of natural myelopoiesis is restored. However, a persistent low strength stimulus during chronic inflammation or cancer causes substantial changes in the biology of these IMCs resulting in their accumulation. During diseased conditions like cancer, the mechanism of emergency myelopoiesis is hijacked by cancer cells, releasing cytokines, chemokines, reactive radicals, immune checkpoint population, and metabolites. These secreted factors strongly contribute to tumor niche formation and to activation of immunosuppressive mechanisms.

The development of MDSC is a complex signalling network which can be understood by dividing the process into two categories: Firstly, signals which promote the accumulation of IMCs and secondly, signals which facilitate pathological activation of IMCs (Condamine et al., 2015a). The accumulation of MDSC in tumor bearing hosts is reported in many clinical studies (Sinha et al., 2008; Porembka et al., 2012; Capietto et al., 2013; Cassetta et al., 2020). Pathological activation of MDSC arises with the persistent stimulation of myeloid cells during prolonged exposure to inflammatory molecules and myeloid growth factors. These activation signals include granulocyte colony stimulating factor (G-CSF), granulocyte-macrophage colony stimulating factor (GM-CSF), macrophage colony stimulating factor (M-CSF), IL-6, IL-1β, VEGF, adenosine, hypoxia-inducible factor (HIF1α) and many more (Gabrilovich and Nagaraj, 2009). Once activated, these MDSC actively participate in various aspects of tumor progression including tumor invasion, angiogenesis, tumor microenvironment formation and epithelial-mesenchymal transition (Karakhanova et al., 2015; Li et al., 2017; Fleming et al., 2018). Furthermore, immune cell suppression is also a defining feature of MDSCs. Despite their profound effects, the characterization standards of MDSC are not very well defined and the defining phenotypic, morphological, and functional features of MDSC are still an area of intensive research.

The major subgroups of MDSCs in humans and mice are granulocytic/polymorphonuclear MDSC (PMN-MDSCs) and monocytic MDSC (M-MDSCs). The classification of PMN-MDSC and M-MDSC is based on their origin in granulocytic and monocytic cell lineages, respectively. Bronte *et al.* in 2016 reported another small group of MDSC in humans termed as early-MDSC (eMDSC) (Bronte et al., 2016). However, the precise characteristic features of the three subtypes of MDSC is still unclear. The three main populations of MDSCs are found at different frequencies in various cancer subtypes and every pathological condition such as cancer, autoimmunity, and infection may affect their existence (Youn and Gabrilovich, 2010). For instance, the fate and differentiation of hematopoietic cells in the bone marrow is affected by tumor cells and is clearly distinct from the effect of infection and sepsis related myelopoiesis. Specifically, the cues that lead to the expansion and mobilization of myeloid cells specially MDSCs, namely, endotoxins, cytokines, chemokines, and hormones, play an important role to define the plasticity and longevity and cytokine profile of MDSCs.

### 2.1 Phenotypic characteristic features of MDSC

In mice, the MDSCs were first characterized as cells expressing Gr-1 and CD11b. These markers were subsequently improved by identifying the existence of subpopulations of MDSCs, namely, PMN-MDSCs (CD11b^+^ Ly6G^+^ Ly6C^low^) and M-MDSC (CD11b^+^ Ly6G^−^ Ly6C^hi^). Ginderachter and others validated the two distinct MDSC subfractions with a detailed description of their morphological, molecular and functional complexity (Movahedi et al., 2008). These cell surface characteristics have defined an initial framework for the MDSC characterization.

With the increased relevance of MDSCs in tumor immunology, uniformity in the classification of MDSC has gained interest. This challenge was explored by the Cancer Immuno guiding Program under the umbrella of the Association of Cancer Immunotherapy to establish the foundation of MDSC phenotypic characteristics. An interlaboratory collaboration of 23 laboratories (15 Europe and 8 US) coordinated a proficiency panel program which aimed at harmonizing the accepted MDSC phenotype. The results documented high variability in MDSC phenotyping which was associated with the variance of gating strategy. Therefore, a strong recommendation to harmonize the marker combinations and gating strategies for the identification of MDSC subsets was proposed (Mandruzzato et al., 2016). This collaborative research successfully described a specific gating strategy for the identification of human MDSC through flow cytometry.

Human peripheral mononuclear blood (PBMC) contains PMN-MDSC which are defined as CD11b^+^ CD14^−^ CD15^+^ or CD11b^+^ CD14^−^ CD66b^+^ cells and M-MDSC defined as CD11b^+^ CD14^+^ CD15^−^ cells. CD33, another myeloid marker can also be used instead of CD11b^+^, since a very few CD15^+^ cells are CD11b^−^. Three markers, namely, Lin^−^ (CD3, CD14, CD15, CD19, CD56), HLA-DR^low^ and CD33^+^ define an immature progenitor of MDSCs. Another immature subset of MDSC is now termed early MDSCs (eMDSC) which are CD11b^+^ and CD33^+^ but CD14^−^/CD15^−^. This group of cells comprises of myeloid progenitors and precursors, representing less than 5% of the MDSC population (Bronte et al., 2016). A recent study also reported a monocytic lineage termed monocyte-like precursors of granulocytes (MLPGs) which differentiates into PMN-MDSCs (Mastio et al., 2019). However, the possibility of differentiating PMN-MDSC from precursor cells requires additional validation.

Despite specific gating criteria for the identification of MDSC (both in human and mouse), discrimination of monocytes from M-MDSC and neutrophils from PMN-MDSCs is difficult because unique phenotypic markers for MDSCs. (Bronte et al., 2016). Functional studies using MDSCs are performed on the entire cell population, therefore the precise nature of M-MDSCs and PMN-MDSCs remains unclear.

Additionally, a major challenge is also observed in recognizing MDSC in tissue samples by immunohistochemistry (IHC). Gr-1 and Ly6G markers are used for identification of MDSC in mice by IHC. However, it is impossible to differentiate between neutrophils and monocytes in frozen and paraffin-embedded tissue samples. Similarly, in human samples, distinction of MDSC from monocytes and neutrophils is difficult. Generally, CD33 is considered for the identification of MDSC in human samples but this does not allow for the discrimination of MDSC from macrophages, dendritic cells (DC) and other myeloid cells. Therefore, a combination of CD33 and S100A9 is suggested for MDSC identification as this excludes DC and macrophages. Some recent studies have also suggested lectin-type oxidized LDL receptor 1 (LOX1; encoded by ORL1 gene) as a specific marker to identify human PMN-MDSCs in peripheral blood and patients with cancer (Condamine et al., 2016; Nan et al., 2018; Kim et al., 2019a; Chai et al., 2019). Similarly, M-MDSC can be distinguished from monocytes by the low expression of MHC class II. The challenge for future studies is the identification of unique cell surface markers of MDSCs so that, their function in various diseases can be explored.

Briefly, in other cancers, evidence suggests that M-MDSCs from tumor bearing (TB) mice can differentiate into PMN-MDSCs, attesting to the transition of monocytic cells to differentiated granulocytes (Youn et al., 2012; Youn et al., 2013). Transcriptional silencing of retinoblastoma (Rb1) gene by histone deacetylase 2 (HDAC-2) has been implicated in this process. Involvement of the Rb1 gene in PMN-MDSC differentiation is also supported by recent preclinical studies in breast cancer (Casbon et al., 2015). However, only a certain percentage of M-MDSCs population can be differentiated into PMN-MDSCs. This evidence raises a discussion as to whether M-MDSCs may constitute granulocytic progenitors. The phenotype and nature of these cells was explored by Mastio and others in 2019. They clearly demonstrated the existence of monocytic precursors of granulocytes which only expanded in mouse models but also in cancer patients. The mechanistic studies showed the myeloid differentiation pathway is controlled by the downregulation of Rb1 which is an important factor for the accumulation of PMN-MDSCs. However, the exact mechanism for abnormal myelopoiesis and reason for expansion of PMN-MDSCs in cancer patients requires additional study.

Phenotypic characteristics of hematological malignancies based on similar patterns of MDSCs appear to be shared by other cancers. For instance, in patients with B cell non-Hodgkin lymphoma (B-NHL) or T-NHL, M-MDSCs were increased as compared to healthy donors, andis associated with advanced stage of lymphoma (Wang et al., 2022). M-MDSC dependent T cell suppression correlated with the overexpression of Arg-1, IL-10, PD-L1 and S100A12 (Lin et al., 2011; Tadmor et al., 2013; Khalifa et al., 2014; Wu et al., 2015; Xiu et al., 2015; Azzaoui et al., 2016). In other lymphoid malignancies an enrichment has been also described in myeloma (Görgün et al., 2013), chronic lymphoid leukemia (CLL) (Jitschin et al., 2014), DLBCL (Tadmor et al., 2013; Azzaoui et al., 2016) and T-cell lymphoma (Movahedi et al., 2008). An expanded immunosuppressive population of CD66b^+^CD33 ^low^ HLA-DR^−^ cells was observed within PBMCs from patients with Hodgkin and non-Hodgkin lymphoma. This was the first report on the presence of G-MDSCs in lymphomas. The study defined the G-MDSCs as CD66b^+^CD33^dim^HLA-DR^−^CD11b^+^CD16^+^ (Marini et al., 2016) and co-related with unfavorable prognosis and disease aggressiveness. M- and G-MDSCs were also reported in peripheral blood of DLBCL patients. Myeloid dependent T-cell suppression was co-related with the release of IL-10 and S100A12 and increase in PD-L1 expression. *In vitro* experiments noted the restoration of T-cell proliferation with the depletion of the M-MDSC population (Azzaoui et al., 2016). In multiple myeloma, MDSCs showed a strong tumor promoting and immune-suppressive activity. Both, M- and G-MDSCs were significantly increased with high expression of ROS and ARG1 in peripheral blood and bone marrow of MM patients as compared to healthy donors (Görgün et al., 2013).

## 3 Functions of MDSC

### 3.1 Immunological function

**FIGURE 1:**
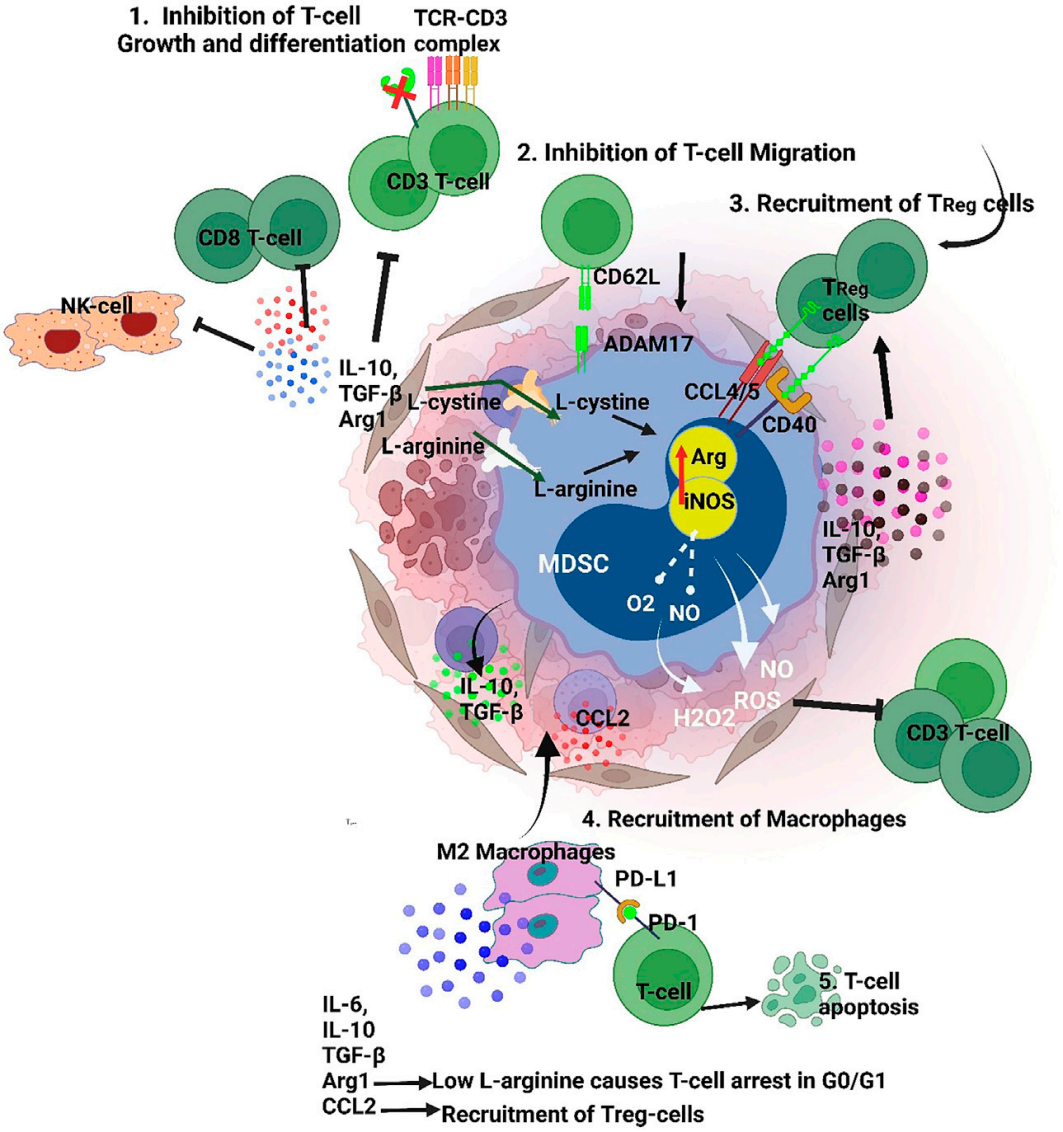
MDSCs suppressive mechanism targets both innate and adaptive immunity 1. Targeting T cell and NK cell function by TGFβ/IL10 induced inhibition. Also, MDSCs release ROS, H_2_O_2_ and peroxynitrite which dysregulate the TCR Z chain inhibiting T-cell/MDSC interaction 2. MDSCs maintain a pre-metastatic niche inhibiting T-cell migration, 3. MDSCs interact with Tregs *via* CCL4/5 and CD40 that recruitments them to the TME, 4. MDSCs induce a M2 macrophage phenotype by secretion of IL-10, thereby promoting immune escape for tumor cells. Furthermore, secretion of factors like IL-10 and TGF-b, and reduction of L-arginine by MDSCs induce Treg polarization. (iNOS-Inducible nitric oxide synthase, Arg- Arginase, PD-L1- Programmed death-ligand 1, TGFβ- Transforming growth factor beta, IL-10- Interleukin 10, 1L-6- Interleukin 6,CCL-2- C-C Motif Chemokine Ligand 2, CCL4- C-C Motif Chemokine Ligand 2, ADAM17-a disintegrin and metalloprotease domain).

The important characteristic that defines MDSC is their suppression of immune cells including B-cells (Li et al., 2014), NK cells (Elkabets et al., 2010; Hongo et al., 2014) and T-cells. Both M-MDSC and PMN-MDSC share similar biochemical features which enables the suppression of the immune response that includes upregulation of signal transducer and activator of transcription 3 (STAT3), expression of Arginase one and S100A8/A9, and induction of ER stress. Specifically, PMN-MDSC upregulate reactive oxygen species (ROS), peroxynitrite, prostaglandin E_2_ (PGE_2_) and arginase 1. Additionally, M-MDSC upregulate nitric oxide (NO), cytokines such as IL10 and TGFβ (Figure 1), and increase expression of PD-L1 (Gabrilovich, 2017). Therefore, MDSCs in the TME strongly contribute to the formation of a pre-metastatic niche and subsequent development of metastatic lesions. MDSCs also facilitate the escape of tumor cells from the primary site by suppressing the immune system *via* multiple signaling pathways which directly corelate to poor patient prognosis. Therefore, MDSC-directed cancer therapies usher in a new era of research for establishing novel anticancer therapies.

#### 3.1.1 T-cell suppressive function

MDSCs affect T-cell function by depleting the fundamental amino acids such as L-arginine and cysteine. Also, MDSC drive ROS production by increasing NADPH oxidase activity. Studies have found that administration of ROS inhibitors effectively regains T-cell activation (Corzo et al., 2009; Wei et al., 2015). The VEGF receptors on MDSC also support expansion and recruitment into theTME (Kusmartsev et al., 2008; Corzo et al., 2009). Other MDSC receptors such as adisintegrinandmetalloproteinase17 (ADAM17), downregulate L-selectin levels on CD4/CD8 T-cells preventing T-cell homing (Hanson et al., 2009). MDSCs have also been shown to increase PD-L1 expression which downregulates T cell mediated antitumor reactivity (Weber et al., 2018). Fuse et al. (2016) effectively blocked PD-L1 and reported a significant decrease in MDSC suppressing T-cell activity (Figure 1). MDSCs produce CCR5 ligands (CCL4 and CCL5) which recruit Tregs and promote immune escape for tumors (Figure 1). Schlecker et al. (2012) have shown that intratumoral injection of CCL4/CCL5 increased tumor infiltration of Treg cells (Figure 1). Similarly, lack of CCR5 reduced the recruitment of Tregs into the TME.

#### 3.1.2 B-cell suppressive function

In 2015, Crook and others reported for the first time B-cell inhibition by MDSCs in a murine model of rheumatoid arthritis (Crook et al., 2015). Despite extensive research, limited studies attest to MDSC and B cell crosstalk. In human PBMC, PMN-MDSCs were shown to modulate B-cell function by suppressing cell proliferation. It was further demonstrated that arginase-1, NO, ROS were involved in the suppression of B-cells (Lelis et al., 2017). A novel finding revealed an interaction between MDSCs and B-cells in tumor bearing mice. It was observed that MDSCs accumulated in the germinal center of the spleens of tumor bearing mice and co-localized with B-cells. *In vitro* co-culture of MDSCs with B-cells promoted proliferation and differentiation of B-cells into plasma cells producing IgA. Further experimental validations suggested that IL-10 and TGF-β1 play an important role in promoting MDSCs mediated IgA production (Xu et al., 2017a).

Wang et al. (2018) and others found that MDSCs suppress B cell function in a murine lung cancer model. It was reported that B-cells were significantly reduced in the bone marrowand spleen of tumor bearing mice. Mechanistic studies revealed that IL-7 and STAT5, key regulators for B cell commitment, were also downregulated. The B-cell impairment was positively co-related with MDSC infiltration and cancer prognosis. Furthermore, IgG levels were decreased with B-cell dysfunction. MDSCs suppressed B-cell proliferation in an arginase-dependent manner, indicating cell-cell interactions between MDSCs and B-cells.

#### 3.1.3 Suppression of other cells

MDSCs are further known to inhibit NK cell cytotoxicity. Expression of indoleamine 2,3-dioxygenase (IDO) expression by MDSCs reduces tryptophan concentration in the TME. This not only stimulates Treg cells (Arandi et al., 2018) but also induces apoptosis of NK cells (Fang et al., 2022). Murine studies have reported MDSC related inhibition of NK cells mediated by TGFβ (Li et al., 2012). Membrane bound TGFβ was shown to impair NK cell cytotoxic activity, reduce NKG2D expression and decrease INF-γ production (Li et al., 2009). Ligands such as IL-33 (Fournié and Poupot, 2018; Shen et al., 2018)- produced by epithelial andendothelial cells and IL-1β (Elkabets et al., 2010) expressed by MDSCs have greater inhibitory properties for NK cells than other immune cells. In humans, MDSC inhibit the effects on INF-γ *via* NКp30-dependent mechanism (Hoechst et al., 2009) and interfere with NK FcR-mediated cytotoxicity which impairs NK cytotoxic and cytokine production (Stiff et al., 2018).

INF-γ and other molecules present in the TME promote MDSC expansion which results in the release of IL-10. The increased amount of IL-10, an anti-inflammatory molecule, inhibits the release of inflammatory cytokines thereby promoting a pro-tumorigenic microenvironment. The IL-10 mechanism strongly suppresses NK-cell and CD8^+^ T-lymphocyte activation (Figure 1) (Ibrahim et al., 2018; Yaseen et al., 2020). Additionally, NO production by MDSCs also arrests NK function and the secretion of INF-γ, granzyme B and TNF-α (Zhang et al., 2018). MDSCs produce IDO, which impairs development and activation of NK cells *via* STAT3 and TGF-β signaling (Sun et al., 2013; Sui et al., 2014). On the other hand, *in-vitro* studies on murine NK cells have shown Jak-3 inhibition and activation of STAT5 when co-cultured with MDSC (Hoechst et al., 2009). Furthermore, other NK-MDSCs cells interactions include TIGIT-CD155 (Sarhan et al., 2016) interactions and IL-1R8 (Molgora et al., 2017) signaling induced by interactions with MDSCs.

### 3.2 Non-immunological function

MDSCs also contribute to the progression of primary tumors by promoting angiogenesis and metastasis. Yang et al. (2004) demonstrated a tumor-promoting role for Gr+CD11b+ cells in tumor bearing animals. Co-injection of tumor cells with Gr+CD11b+ cells increased angiogenesis, reduced tumor necrosis and enhanced tumor growth. It was also reported that MMP9 produced by Gr+CD11b+ cells contributes to tumor angiogenesis and growth. In another study, MDSCs isolated from mouse tumors displayed STAT3 activation and induced angiogenesis by expressing VEGF (Kujawski et al., 2008). MDSCs also promote a mesenchymal phenotype in tumor cells by secreting several inflammatory factors such as TGF-β, IL-6, IL1-β and Hepatocyte Growth Factor (HGF) (Figure 1). These factors reduce E-cadherin expression in tumor cells which drives epithelial-mesenchymal transition (Toh et al., 2011; Ouzounova et al., 2017; Pastaki Khoshbin et al., 2019).

Studies in the literature have reported a critical role for MDSCs in establishing a pre-metastatic niche. Generally, primary tumors release signaling molecules before the onset of metastasis to regulate secondary tumor formation and recruitment of cells including neutrophils, macrophages, T_reg_ and MDSCs. These cells communicate *via* signals that prepare healthy tissues at secondary sites to act as “soil” that encourage the inhabitation of circulating tumor cells (CTCs) (Peinado et al., 2017; Nasrollahzadeh et al., 2020; Wu et al., 2020). It has been reported that VEGFR1^+^ hematopoietic bone marrow progenitors promote the formation of a pre-metastatic niche that supports tumor metastasis. VEGFR1^+^ cells express VLA-4 (also known as integrin alpha4beta1) which can act in concert to promote a supportive niche for tumor growth (Kaplan et al., 2005). A study by Yan et al. (2010) reported an increased population of Gr-1+CD11b+ cells (immature myeloid cells) in the lungs of mice bearing mammary adenocarcinoma. The immature myeloid cells in the premetastatic lungs showed significant decrease in INF-gamma production with increased production of proinflammatory cytokines. Also, these cells expressed high levels of matrix metalloproteinase 9 (MMP9) and promoted vascular remodeling. The deletion of MMP9 diminished lung metastasis. Further studies have shown MDSC derived exosomes, containing TGF- β, VEGF and S100A8/A9, significantly induced metastasis, immunosuppression and promotes PMN-MDSC expansion (Wang et al., 2019a; Hsu et al., 2019).

### 3.3 Gene expression profiles of MDSC from solid tumors

The MDSCs genotype has benn widely studied in several cancers, namely, breast cancer, colorectal cancer, lung adenocarcinoma, etc. However, research on hematological malignancies is much more limited as indicated by gaps in the literature. For instance, an RNA sequencing analysis on non-small cell lung carcinoma (NSCLC) patients by Mastio and others defined of M-MDSCs and monocytes with distinct gene signature profiles. The gene expression profile of HLA-DR^−/LOW^ CD14^+^ CD15^−^ showed 619 differentially expressed genes as compared to monocytes. A notable upregulation of neutrophil-associated genes was observed including IL-8, MNDA (Myeloid cell nuclear differentiation antigen), CSF3R (G-CSF receptor), LYZ (lysozyme), NCF4 and NCF2 (neutrophil cytosolic factors 4 and 2). Additionally, downregulation of CSF1R (M-CSF receptor), NR4A1 (Nur77), CXCR1, ITGA4 (CD49d) and CD52 was observed (Table 1) (Mastio et al., 2019). The higher expression of neutrophil function genes in HLA-DR^−/LOW^ CD14^+^ CD15^−^ cells suggests that these cells may have neutrophil precursors. Further extensive analysis showed an upregulation of the *CXCR1* gene, which is a chemokine receptor on neutrophils and is not associated with monocytes. Experimental validation on prostate cancer in the same study showed that 20% of M-MDSCs were *CXCR1* positive and had potent immunosuppressive activity. Furthermore, these CXCR1^+^ M-MDSCs not only had the ability to differentiate into neutrophils but also shared features of macrophages (Mastio et al., 2019). This study therefore strongly attests to the enrichment of MLPGs (MDSC originating from M-MDSC and differentiating into PMN-MDSC) in humans.

**TABLE 1:** Phenotype and genotype of MDSCs in human cancer.

MDSCs	Tumor entity	Sample type	Phenotypic markers	Genotypic markers
PMN-MDSCs (Lin^−^ HLA^−^DR^-^ CD11b^+^ CD33^+^ CD15^+^/CD66b^+^	NSCLC, head and neck cancer	PB	LOX1 Condamine and Gabrilovich. (2011); Condamine et al. (2016)	
	NSCLC	PB	FATP2 Veglia et al. (2019)	
	Head and neck cancer	PB	DR5 Condamine et al. (2014)	
	Breast cancer	PB	CD84 Alshetaiwi et al. (2020)	WFDC17, CD84, ARG2, IL1B Alshetaiwi et al. (2020)
	Multiple myeloma	BM		PTGS2, CSF1, IL-8, IRF1, IL4R, STAT1, STAT3 and STAT6, TGFB1 Perez et al. (2020)
	NSCLC	PB		AQP9, LILRA5, CXCL3, BCL2A1, LILRA6, C19orf59, LIRRB2, CCL18 LYN, CCL20 MEFV, CD300E MMP12, CTSC NCF2, CXCL1 OSM, CXCL2 PLAUR, CXCL3 PTAFR, CXCL5 S100A12, CXCL6 S100A8, CXCR1 S100A9, CXCR2 SERPINB8, EMR2 SLC11A1, EMR3 SOD2, FCGR3B TREM1, FGR, FPR1, GPR97, HCK, HK3, ICAM1, IL15RA, IL1B, IL1R2, IL1R2, IL6, IL2RA, IL8, LILRA3, TREM1 Veglia et al. (2021)
	NSCLC	Lung cancer tissue		IL6, OLR1, and TGFB1
	MM	BM and PB	ROS, ARG Görgün et al. (2013)	
M-MDSC (Lin^−^ HLA^−^DR^-^ CD11b^+^ CD33^+^ CD14^+^	MM	BM and PB	ROS, ARG Görgün et al. (2013)	
	B-cell non-Hodgkin lymphoma	PB	Arg-1, IL-10, PD-L1 and S100A12 Lin et al. (2011); Tadmor et al. (2013); Khalifa et al. (2014); Wu et al. (2015); Xiu et al. (2015); Azzaoui et al. (2016)	
	DLBCL	PB	IL-10 and S100A12 Azzaoui et al. (2016)	
	Head and neck cancer	PB	DR5 Condamine et al. (2014)	
	Ovarian cancer	Blood, ascites, and tissue	PD-L1 Okła et al. (2020)	PD-L1 Okła et al. (2020)
	NSCLC	PB		S100A8, S100A9 Feng et al. (2012)
	Breast cancer	PB	CD84 Alshetaiwi et al. (2020)	WFDC17, CD84, ARG2, IL1B Alshetaiwi et al. (2020)
	NSCLC	Lung cancer tissue		IL10, CD14, and VEGFA
	Melanoma	Spleen	IL-6, IL-8, Tobin et al. (2019)	CXCR1

NSCLC, Non-Small Cell Lung Cancer; PB, peripheral blood; BM, bone marrow; LOX1, Lectin-like oxidized low-density lipoprotein (LDL) receptor-1; FATP2, Fatty acid transport protein; DR5, Death receptor 5; WFDC17, whey acidic protein-type four-disulfide core domain 17; ARG2, Arginase 2; IL1B, Interleukin-1beta; PTGS2, Prostaglandin-endoperoxide synthase; CSF1, Colony Stimulating Factor 1; IL-8, Interleukin 8; IRF1, Interferon Regulatory Factor 1; IL4R, Interleukin four Receptor; STAT1, Signal transducer and activator of transcription 1; STAT3, Signal transducer and activator of transcription 3; STAT6, Signal transducer and activator of transcription 6; TGFB1, transforming growth factor-beta; AQP9, Aquaporin 9; LILRA5, Leukocyte Immunoglobulin Like Receptor A5; CXCL3, Chemokine (C-X-C motif) ligand 3; BCL2A1, BCL2 Related Protein A1; LILRA6, Leukocyte Immunoglobulin Like Receptor A6; C19orf59, chromosome 19 open reading frame 59; LIRRB2, Leukocyte Immunoglobulin Like Receptor B2; CCL18, C-C Motif Chemokine Ligand 18; LYN, LYN, proto-oncogene; CCL20, C-C Motif Chemokine Ligand 20; MEFV, MEFV, innate immunity regulator, pyrin, MMP12, (Matrix Metallopeptidase 12; CTSC, Cathepsin C; NCF2, Neutrophil Cytosolic Factor 2; OSM, Oncostatin M; PLAUR, plasminogen activator, Urokinase Receptor; PTAFR, platelet activating factor receptor; CXCL5, C-X-C Motif Chemokine Ligand 5; S100A12, S100 Calcium Binding Protein A12; S100A9, S100 Calcium Binding Protein A9; CXCR2, C-X-C Motif Chemokine Receptor 2; SERPINB8, Serpin Family B Member 8; EMR2, EGF-Like Module-Containing Mucin-Like Hormone Receptor-Like 2); SLC11A1, Solute Carrier Family 11A Member 1; EMR3, EGF-like module-containing mucin-like hormone receptor-like 3; SOD2, Superoxide Dismutase 2; FCGR3B, Fc Gamma Receptor IIIb; TREM1, Triggering Receptor Expressed On Myeloid Cells 1; FGR, FGR, Proto-Oncogene, Src Family Tyrosine Kinase; FPR1, Formyl peptide receptor 1; GPR97, G protein-coupled receptor 97; HCK, HCK, Proto-Oncogene, Src Family Tyrosine Kinase; HK3, Hexokinase 3; ICAM1, Intercellular Adhesion Molecule 1; IL15RA, Interleukin 15 Receptor Subunit Alpha; IL1B, Interleukin-1beta; IL1R2, Interleukin one Receptor Type 2; IL6, Interleukin 6; IL2RA, Interleukin two Receptor Subunit Alpha; LILRA3, Leukocyte Immunoglobulin Like Receptor A3; TREM1, Triggering Receptor Expressed On Myeloid Cells 1; TGFB1, Transforming Growth Factor Beta 1; ROS, Reactive oxidation species; ARG, Arginase; DLBCL, Diffuse large B-cell lymphoma; MM, multiple myeloma.

Another study on pancreatic ductal carcinoma (PDAC) showed distinct cytological features (small size) and T cell suppressive function in M- MDSCs. Genome wide mRNA expression profile on M-MDSCs from three suppressive and four non-suppressive PDAC patients demonstrated an overall cancer-related gene signature including increased TNFα signaling *via* NF-κB, inflammatory response, IL6 JAK/STAT3 signaling and apoptosis categories. The suppressive CD14^+^ cells showed higher expression of FBN2, TSPAN16, LEPR, CLTA and CD163 which are genes associated with classic monocytes and differential expression patterns of MAP3K3, PRKRA, JAK2, as well as differential expression of components of the STAT family (STAT1, STAT2, STAT3, STAT5A, STAT5B and STAT6) (Trovato et al., 2019). This study also reported upregulation of metabolic genes linked to immunosuppression including fatty acid and lipoprotein metabolism-related genes, namely, CD36, LYPLA1 and CERS5, ATP and glucose metabolism genes such as ATP51C, ATP5G2, SDHB and PDK4, GXYLT1. Additionally, the BM-MDSCs and cancer patient immunosuppressive M-MDSCs shared a common gene signature profile, i.e., non-differentially expressed genes such as PTGS2, TNF, CD38, ARG1, AKT3, JAK1, JAK3, STAT1, STAT4, STAT5, STAT6 and STAT3, suggesting a common regulatory mechanism among the myeloid cells. Briefly, this study states an important immunosuppressive phenotype of M-MDSCs (Table 1).

Other studies have also reported CD33 as a specific marker expressed on MDSCs (Ortiz et al., 2015), which is one of the potential targets for cancer therapies (Jitschin et al., 2018). Other genes associated with gene ontology (GO) analysis included chemokine receptor 2 (CXCR2) (Veglia et al., 2019; Ugolini et al., 2020), colony Stimulating Factor 1 Receptor (CSF1R) (Zhou et al., 2018), and C-C Motif chemokine receptor 1(CCR1) (Cai et al., 2017), suggesting that MDSCs might communicate with recruitment signals from other sites of inflammation including the primary tumor or metastatic sites. This study also validated MDSC gene signatures from spleens of healthy and tumor bearing mice the upregulation of CD11B + Gr1+ cells (Alshetaiwi et al., 2020).

Leader et al. applied scRNA to corelate transcriptome profiles of tumor early-stage NSCLC and normal tissues from early-stage NSCLC. The interrogation into immune ecosystem revealed a remarkable heterogeneity and plasticity in the myeloid cells. According to analyses, M-MDSCs trajectory analysis M-MDSCs and PMN-MDSCs transitioned along the monocyte-to-M2 macrophage path. However, MDSCs subsets did not show any root or branch-level enrichment. Gene expression analysis of M-MDSCs and PMN-MDSCs showed a distinct gene signatures of IL-10, CD14, VEGFA and IL-6, OLR1, TGFβ1, respectively (Leader et al., 2021).

Studies in the literature attest to distinct transcriptome profiles of MDSCs with the upregulation of genes characterized by immunosuppression, high transcriptional activity, and increased pro-inflammatory pathways. These hallmark genes can be further explored in hematological malignancies and their functions can be studied. However, we also face challenges to clearly distinguish MDSCs from monocytes and neutrophils. Therefore, a more comprehensive analysis the gene expression signatures of the MDSCs subtypes will create new opportunities for selective targeting MDSCs populations and thereby designing new therapies.

## 4 MDSCs in hematological malignancies

Mostexperimental and clinical studies on solid tumors have reported dysregulation of MDSCs However, recent studies have defined their role in immune dysregulation in hematologic malignancies, immune-mediated cytopenias and allogeneic hemopoietic stem cell transplantation. Also, MDSCs potential role as biomarkers and therapeutic targets have started gaining attention in B-cell malignancies. The signaling pathways associated with the differentiation, expansion, circulation, and function of MDSCs, as well as the crosstalk between MDSCs and tumor cells make therapeutic strategies challenging. This section will summarize the research studies on the MDSCs in hematological malignancies and their tumor microenvironment.

### 4.1 MDSCs in tumor microenvironment

The TME of B-cell lymphomas is highly variable, which consists of immune cells, stromal cells, blood vessels and extracellular matrix. Genetic aberrations harbored by tumor cells, their proliferation, increased angiogenesis, and an immune response by the host, contributes to the complex spatial cellular distribution of TME. Therefore, crosstalk between the TME elements is distinct in different types of lymphoma. Lymphomas can have variable amounts of tumor cell content, which ranges from a minimum of ∼1% in Hodgkin’s lymphoma to a maximum of 90+ % in Burkitt’s lymphoma (BL). Generally, the TME in hematological malignancies can be divided into three models based on the interaction between tumor cells and the TME: 1. ‘recruitment’, 2. ‘re-education,’ and 3. ‘effacement.’

Briefly, the ‘re-education’ pattern is commonly seen in FL, CLL, MM, and MALT lymphomas, which primarily affect lymph nodes. In this pattern, the malignant cells have a substantial degree of dependency on the TME for survival. Proliferation of lymphoma cells is partially supported by external stimuli in the TME, such as cytokines, chemokines, growth factors and signaling *via* stromal cells. The spatial arrangement of the tumor tissue is very similar to the normal lymphoid tissue, comprising of residual reactive germinal centers with distinct follicular dendritic cell (FDC) meshworks and follicular T-helper cells (T_FH_ cells) (Scott and Gascoyne, 2014; Nathan et al., 2016).The second pattern is one of ‘recruitment’ and is typically observed in classical Hodgkin’s lymphoma (CHL). Here, the tumor comprises of a supportive non-malignant milieu which surrounds the infrequent malignant Hodgkin Reed-Sternberg cells (HRS cells). In this pattern, the tumor cells rely completely on the TME for survival and growth (Scott and Gascoyne, 2014; Nathan et al., 2016). Lastly, the third pattern is ‘effacement’ and includes BL where malignant cells express genetic aberrations such as MYC translocations and cooperative mutations (Schmitz et al., 2012). These changes remove the dependency of the malignant cell on the microenvironment for growth and proliferation, causing cell-autonomous survival (Scott and Gascoyne, 2014; Nathan et al., 2016).

As discussed earlier, the composition of TME in B-cell malignancies in divided into two parts: Immune microenvironment and non-immune microenvironment. The immune microenvironment consists of immune cells including T and B lymphocytes, MDSCs, tumor-associated macrophages (TAMs), tumor associated neutrophils (TANs), NK cells, dendritic cells, and many other cells. Furthermore, the non-immune microenvironment mostly includes fibroblasts, stromal cells, secretory molecules including growth factors, exosomes, cytokines, and chemokines.

TAMs are the most intensive immunosuppressive cells in TME. These cells inhibits T-cell activation and recruits at the tumor site by secreting cytokines, chemokines and other factors (Pathria et al., 2019). Based on immunomodulatory functions, TAMs are classified into M1 and M2 phenotypes. M1 macrophages are cytotoxic and play a critical role in tumor development. However, during the process of tumor growth M1-like macrophages are transformed into M2-like macrophages (Mantovani et al., 2017). M2-like macrophages produce cytokines and growth factors which contribute to tumor development, angiogenesis, inflammation and immunosuppression (Wang et al., 2019b) (Figure 1). As discussed in previous sections, the TME cells express specific cell surface markers which is associated with poor prognosis in B cell lymphoma patients. The cytokines and chemokines secreted by TME cells creates an immunosuppressive environment which modulates the activity of immune cells such as suppression of T cell function. These cytokines are divided into two groups. The first group includes VEGF, granulocyte-macrophage colony-stimulating factor (GM-CSF), macrophage colony-stimulating factor (M-CSF) and granulocyte colony-stimulating factor (G-CSF).Similarly, the second group of cytokines includes interferon-γ (INF- γ), IL-6, IL-4, IL13, high-mobility group box 1 (HMGB1), and tumor necrosis factor (TNF) (Sevko et al., 2013; Condamine et al., 2015b).

A direct relationship between MDSC and TME is reported in many cancers including prostate, breast, colon, melanoma and in B-cell malignancies. As an important immunosuppressive cell, MDSCs effectively suppress T-cell, B-cell, and NK cell activation, and recruits Tregs at the site of tumor. Therefore, tumor cells escape by the immune surveillance and increases tumor growth and metastasis. A recent report demonstrated the secretion of exosomes in TME. These tumor derived exosomes accelerate the activation and expansion of MDSCs (Tian et al., 2019) delivering molecules and proteins such as miRNAs (Ren et al., 2019), IL-10, 1L-16, PEG2 and TGF-β.

### 4.2 Lymphomas

A preclinical study on murine A20 B-cell lymphoma model, Serafini and others identified a population of cells with a unique phenotype. These cells showed expression of Gr1, F4/80 and IL-4Rα, with low expression of MHC class I and II molecules. In subsequent studies, the population was confirmed to be MDSCs, and these cells suppressed CD8^+^ T cell activity and induced recruitment and expansion of Treg cells (Serafini et al., 2008) (Figure 1). The immunosuppressive activity of MDSCs was inhibited by lenalidomide, in that the drug reduced the frequency of MDSCs in A20 lymphoma mice. (Sakamaki et al., 2014). Furthermore, inflammatory changes promoted infiltration of MDSCs in the spleens of EL4-luc2 lymphoma mice model. These mice demonstrated elevated numbers of neutrophils resulting in abnormal myelopoiesis, which stimulated MDSCs thereby supporting tumor development (Abedi-Valugerdi et al., 2016). In murine B- cell lymphoma, the expression of mir-30 further promoted the development of MDSCs. Activation of JAK/STAT3 signaling pathway by miR-30 decreased the expression of suppressor of cytokine signaling-3 (SOCS3), thereby promoting differentiation of MDSCs and releasing immunosuppressive factors such as ARG-1, IL-10 and ROS (Xu et al., 2017b). To confirm increased levels of PDL-1 expression and expansion of MDSCs (at the tumor site) in DLBCL patients, PDL-1 expression was evaluated in an A20 B-cell lymphoma mouse model. It was observed that anti-PDL-1 treatment decreased MDSCs at the tumor site (Lu et al., 2021). Further, another study identified calmodulin kinase 2 (Camkk2) as an important target responsible for the accumulation and expansion of MDSCs in, E.G.,7-OVA TB mice (a mouse lymphoma model derived by electroporating T lymphoblast, EL4 cells). Knockdown of Camkk2 in mice decreased the accumulation of MDSCs, enhanced the anti-tumor response of T-cells and slowed tumor growth. Tumor growth was restored in Camkk2^−/−^ mice when transplanted with MDSCs. This study confirmed the immune suppressive function of MDSCs and potential role of Camkk2 in targeting MDSCs expansion (Huang et al., 2021). Also, M-MDSC accumulation *via* an IL-35 mechanism was significantly decreased with the anti-IL-35 treatment in the Ly8DLBCL murine model (Wang et al., 2021a).

Clinically, Romano et al. (2015) reported an increase in M-MDSCs, PMN-MDSCs and CD34^+^ MDSCs in peripheral blood of Hodgkin’s lymphoma (HL) patients as compared to healthy individuals. It was observed that the levels of MDSCs were much lower in complete remission patients when compared to the patients who did not have a complete remission after chemotherapy. Amini et al. (2019) also observed increased numbers of PMN-MDSCs in the peripheral blood of 19 HL patients when compared to healthy individuals. A similar study found that PMN-MDSCs (CD66b^+^ CD33^dim^ HLADR^−^) were higher in 124 B-cell lymphoma patients which included HL and B-NHL. The study found that depletion of CD66b^+^ MDSCs restored the T-cell response and proliferation (Marini et al., 2016). Furthermore, in DLBCL patients, there was a significant increase in MDSCs in the peripheral blood and this was a poor prognostic factor. However, only the increased number of M-MDSCs correlated with the international prognostic index (IPI), high numbers of circulating Treg cells and elevated expression of IL10, PDL-1 and S100A12. These factors were associated with the immunosuppressive mechanism of MDSCs, and inhibition of IL-10, PDL-1 and S100A12 increased T-cell activation and proliferation (Azzaoui et al., 2016). Some recent studies have reported that high levels of M-MDSCs in newly diagnosed and relapsed patients correlated with tumor progression and patient survival. Additionally, a clinical trial with lenalidomide and R-GDP (Rituximab plus gemcitabine, cisplatin and dexamethasone) chemotherapy in relapsed DLBCL patients showed decreased levels of MDSCs and Treg cells in individuals with a favorable 24-month survival rate. This study also demonstrated that Vit-D deficient DLBCL patients had increased numbers of MDSCs and Treg cells, suggesting that an enhanced treatment outcomes could be achieved by Vit-D supplementation in DLBCL-patients (Jiménez-Cortegana et al., 2021a; Jiménez-Cortegana et al., 2021b). Additionally, patients with NK/T-cell lymphoma (ENKL) had high MDSCs levels and specially M-MDSCs were responsible for a poorer disease-free survival and overall survival. Studies have found that IL-17 produced by CD4+Th17 cells in ENKL patients increases MDSC expansion and inhibits T cell proliferation (Zhang et al., 2015). The effect of MDSCs on other types of NHL including cell lymphoma and follicular lymphoma is still unexplored.

### 4.3 Multiple myeloma

MDSCs expansion in bone marrow was observed during early stages of multiple myeloma (MM) in the 5TMM mouse model and in later stages, MDSCs could be detected in peripheral blood. Also, myeloid leukemia cell differentiation protein (Mcl-1) was reported as a potential target promoting MDSCs survival. *In vitro* studies have shown that mesenchymal stem cell (BMSC)- derived exosomes induced the survival of MM MDSCs and increased NO production in MDSCs by activating STAT3 and STAT1 signaling. These bone marrow-derived mesenchymal stem/stromal cells (BMSC) also increased the expression of anti-apoptotic proteins, namely, Bcl-xL and Mcl-1 suppressing T-cell activation and contributing to bortezomib and melphalan resistance (Wang et al., 2015a; Veirman et al., 2015; De Veirman et al., 2019). A mechanistic study reported the presence of S100A9 and its receptor TLR4 in MM MDSCs model mice which triggered MDSCs expansion and secreted inflammatory molecules such as TNF- α, IL-6 and IL-10 (Figure 1). However, blocking S100A9 did not affect the MDSCs expansion but rather decreased the cytokine production (De Veirman et al., 2017). Further, in a clinical study, a pro-tumorigenic affect of MM derived Galectin-1 was observed on the expansion of M-MDSCs with CD304 interaction, thereby facilitating MM progression following autologous stem cell transplant (ASCT) (Lim et al., 2021). These studies attest that MDSC in the MM microenvironment play an important role in interaction between cells, cytokines and other factors present in the tumor niche which together influence MM progression.

Clinically, for the first time in 2010, Brimnes et al. (2010) reported an increase in M-MDSCs in peripheral blood of MM patients as compared to controls. In later studies Wang et al. (2015b) showed a positive correlation of M-MDSCs with MM recurrence and negative correlation with treatment outcomes. A similar study found out that neutrophils from bone marrow were immune suppressive and had MDSCs like activity. Furthermore, it was found that CD11b^+^ CD113^+^ CD16^+^ neutrophils have G-MDSCs (Perez et al., 2020) phenotype.

Several studies have reported an accumulation of G-MDSCs in the bone marrow and peripheral blood of MM patients as compared to healthy individuals and the expansion is corelated with the disease pathogenesis (Ramachandran et al., 2013; Favaloro et al., 2014; Giallongo et al., 2016). In context to treatment with bortezomib, lenalidomide (immunomodulatory agent) and DC vaccination there are inconsistent results on its effect on MDSCs and MM patients (Gunes et al., 2020). A study by Kuwahara-Ota et al. (2020) identified lenalidomide and pomalidomide as potential inhibitors for MDSCs induction, which downregulated CCL5 and MIF in MM patients. This study also found out that downregulation of CCL5, and induction of interferon regulatory factor 8 (IRF8) affected MDSCs expansion. Additionally, it was also observed that high amount of M-MDSCs in MM patients inhibited the cytotoxicity of pre-autologous stem cell transplantation (ASCT) and was associated with poor clinical outcome (Lee et al., 2019). A co-culture study of MPC11 cells (B lymphocyte cell line) with MDSCs of MM patients showed high proliferation of MPC11 cells and reduction in T cell immune response when compared to no MDSCs, and decitabine (DAC) treated bone marrow cells (Zhou et al., 2019).

A study by Nakamura et al. (2018) have reported the high expression of IL-18 in the bone marrow microenvironment of MM patients. It was observed that, IL-18 driven MDSCs immune suppression *via* C/EBβ was associated with poor prognosis. The migratory inhibitor factor in the MM bone marrow microenvironment is an important factor which leads to the expansion and differentiation of MDSCs, increases levels of PDL-1 expression on MDSCs and suppresses T-cell function (Lewinsky et al., 2021). But now we know that some MM targeting drugs such as Daratumumab (targeting CD38) successfully diminished the immune suppressive cells, namely, Tregs, Bregs and MDSCs (van de Donk and Usmani, 2018). Additionally, estrogen has also been associated with MM disease progression (Ozerova and Nefedova, 2019). Together, all the studies identified new molecules which promotes MDSC formation/expansion and targeting them could improve drug therapeutics for MM.

### 4.4 Leukemia

In earlier studies, Sun et al. (2015) found expansion of MDSCs in acute myeloid leukemia (AML) and a complete remission group of patients as compared to partial and no remission group of patients. This study also reported the overexpression of WT1 (Wilms tumor gene) gene in the bone marrow samples of AML patients which had positive correlation with MDSCs expansion. Another study identified higher amounts of M-MDSCs and e-MDSCs in the peripheral blood of AML patients (Lv et al., 2021). Further, an elevated expression of MDSCs corelated with the poor remission, higher relapse rates and reduced long term survival (Wang et al., 2020).

In AML, it was also observed the cytarabine (Ara-C)- triggered increased expression of TNFα from the AML cells resulting in the expansion and survival of the MDSC by activating IL-6/STAT3 and NFКB signaling (Bai et al., 2022). It has also been observed that the combination of Ara-C, Plerixafor (CXCR4 inhibitor) and PDL-1 in AML murine model decreased the number of Tregs and MDSCs in peripheral blood and bone marrow (Hwang et al., 2019). Additionally, MDSC expansion was observed in B-cell acute lymphoblastic leukemia (B-ALL) (Zahran et al., 2021). Hohtari et al. (2019) noted that in all the AML bone marrow (n = 52) there was a decrease in M1-type macrophages and effector T cells with an increase in M2-type macrophages and MDSCs as compared to healthy individuals. They also observed an elevation of PD-1 and CTLA-4 (negative immune check point) which was responsible for immune suppressive role in AML patients. Furthermore, in acute promyelocytic leukemia (APL) patients innate lymphoid cells (ILC2s) were increased and hyper-activated which in turn activated M-MDSCs *via* IL-13 secretion. After treatment with all trans retinoic acid (ATRA), the levels of PGD2, NKp30, ILC2s, IL-13 and M-MDSCs was restored (Trabanelli et al., 2017).

In chronic granulocytic leukemia (CML), an increased number of MDSCs and its immune suppressive markers such as IL10 and ARG1 was observed. Further, it was noted that imatinib and dasatinib (tyrosine kinase inhibitor) treatment reduced MDSCs numbers to the normal range (Giallongo et al., 2014; Christiansson et al., 2015). Additional studies also reported the tyrosine kinase inhibitor (TKI) treatments reduced G-MDSCs in patients, however, only dasatinib showed a significant reduction in M-MDSCs. Therefore, dasatinib can serve as an important targeting agent for M-MDSCs in chronic myeloid lymphoma (CML) patients (Giallongo et al., 2018). Also, an elevated number of M-MDSCs was observed in the peripheral blood of fifty CLL patients and was associated with poor survival (Zahran et al., 2020). It was also seen that Tregs and MDSCs can be reduced to normal range with ibrutinib treatment in 1–2 years (Solman et al., 2021). Furthermore, PMN-MDSCs showed greater immunosuppressive impact on M-MDSCs in CLL patients. However, ibrutinib successfully lowered the PMN-MDSCs, targeted MDSCs differentiation, induced naïve T cells and improve the tumor microenvironment (Ferrer et al., 2021).

### 4.5 Myeloproliferative syndromes

The first study on MDSC expansion in myeloproliferative syndromes (MDS) was shared by Chen et al. (2013). Using multiple cell transfection models, it was noted that the expansion of MDSCs was driven by S100A9 and CD33. These two important proteins form functional ligand/receptor pairs which suppress immunoreceptor tyrosine-based inhibition motif and induce secretion of suppressive cytokines such as IL-10 and TGFβ. In MDS, MDSCs suppress T cells by recruitment and proliferation of Tregs in the bone marrow (Kittang et al., 2016). Also, Gal-9, an important ligand for immune checkpoint molecule TIM3 is highly expressed on the MDSCs of MDS patients. This Gal9 was observed to bind TIM3 on CD8+T cells, suppressing their immune function and cause T-cell exhaustion (Tao et al., 2020). Studies have also shown that ARG1, an immune suppressive molecule suppresses the ani-tumor response of CD8^+^ T cells *via* STAT3 signaling pathway (Qi et al., 2021) (Figure 1). Therefore, targeting these negatively corelated immune checkpoint molecules of MDSCs in MDS patients could suppress the activation and proliferation of MDSCs.

### 4.6 Hematopoietic stem cell transplantation

**FIGURE 2:**
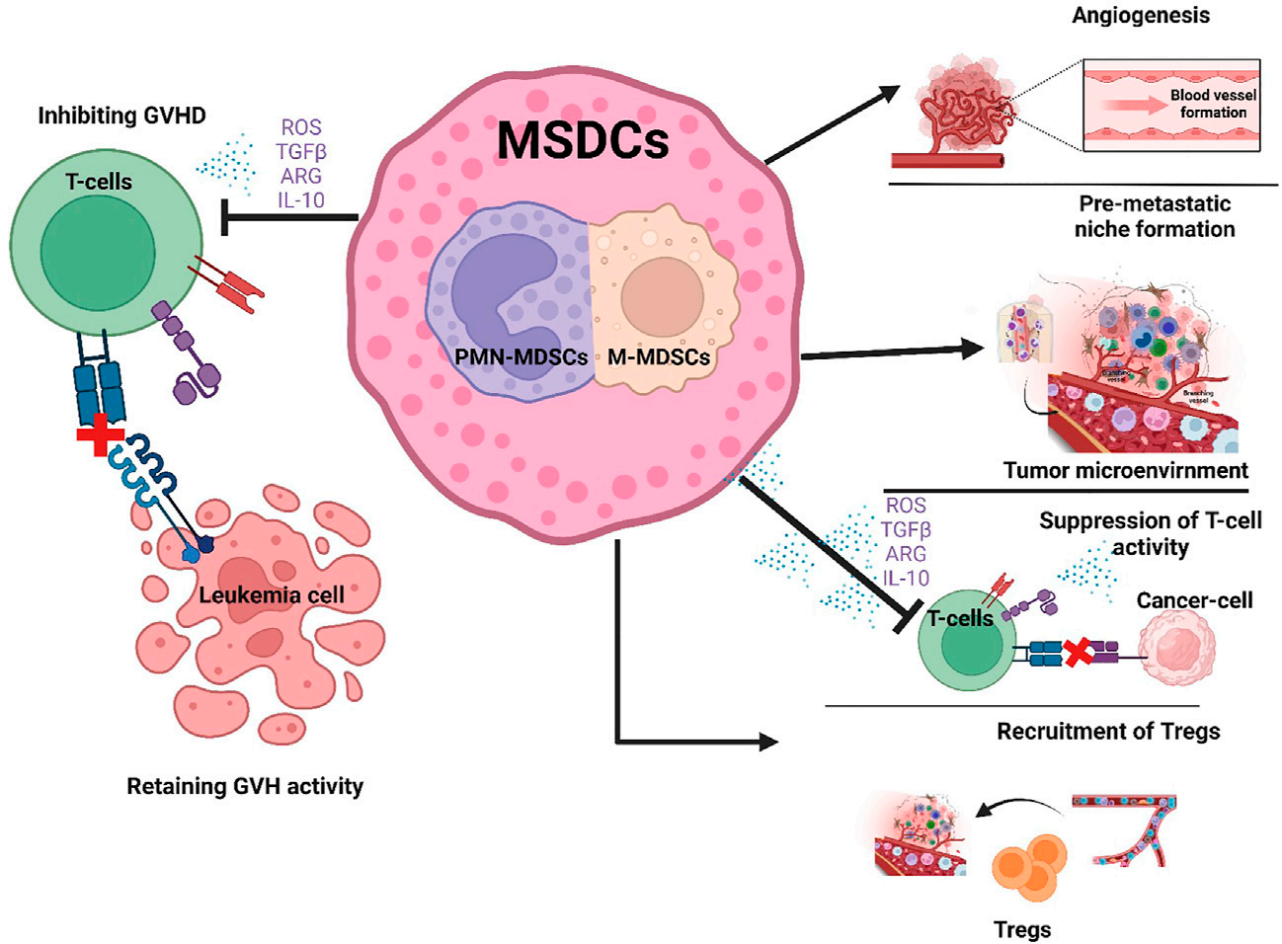
Role of MDSCs in hematological malignancies and Hematopoietic stem cell transplantation. MDSCs have a favorable role in that they suppress T cell activity thereby preventing GVHD after HSCT (Left side of figure). In contrast, the unfavorable expansion of MDSCs promotes tumor growth by increasing angiogenesis, creating a pre-metastatic niche, suppressing T-cell activity and recruiting Tregs in the TME (Right side of figure).

In previous sections we have discussed the immune suppressive and tumor promoting role of MDSCs in hematological malignancies. However, in hematological stem cell transplantation (HSCT), the relationship between graft versus leukemia (GVL), graft versus host disease (GVHD) and MDSCs is complicated (Figure 2).

In mice and humans, the expression of MDSCs have been detected in the peripheral blood during allogenic hematopoietic stem cell transplantation (allo-HSCT) (Luyckx et al., 2012). A clinical study by Fan et al. (2017) showed that G-CSF mobilization improved relapse free survival and GVHD in bone marrow hematopoietic transplantation (G-BM) as compared to G-PBMC transplantation. A similar study also showed that mobilization with pegylated-G-CSF in allo-HSCT elevates M-MDSCs in the donors and improved the rates of severe GVHD (Li et al., 2021).

A transcriptomic study by Andrea et al. reported that G-CSF mobilized PMN-MDSCs had upregulation of genes promoting DNA replication, cell cycle, and cell division, namely, Ki-67, topoisomerase II alpha, and cyclin B, respectively. This study showed that G-CSF-mobilized PMN-MDSCs enrichment in the graft displayed remarkable molecular characteristics with significant inhibition of donor NK cells (Pelosi et al., 2021). A retrospective study reported encouraging improvement in ani-leukemic affect of donor lymphocyte infusion (DLI) after G-CSF treatment as compared to standard DLI after relapsed allo-HCT (Schneidawind et al., 2019). Wang et al. (2021b) described that human umbical cord mesenchymal stem cells causes MDSCs expansion and prevents GVHD after HSCT by secreting CXCL1 and HLA-G (Yang et al., 2020). The mechanistic study on GVHD mice models revealed that MDSCs suppresses GVHD and preserves GVL activities by inducing NKG2D expression on T cells and suppresses GVHD by increasing Tregs (Zhang et al., 2019). It was also observed that the delay in M-MDSCs recovery and invariant natural killer cells (iNKT) after transplantation was associated with a high incidence of grade III-IV acute GVHD. However, low levels of M-MDSCs and high levels of iNKT cells significantly reduced leukemic relapse (Kim et al., 2019b). This study suggests that the balance between MDSCs and other immune cells is critical for achieving good outcomes in HSCT.

The major challenges in the field of GVHD during transfusion of immune suppressive cells is the inefficient immune system, dysregulation in growth of immune cells and the risk of infection. Pre-transplantation measures together with stem cell infusion and inflammation in the host causes M-MDSCs expansion, and this expansion of MDSCs causes non-relapse mortality (Lee et al., 2018). In recent studies treatment with cyclophosphamide (an immunosuppressant) after allo-HSCT caused early differentiation of MDSCs with reduced GVHD incidence (D'Aveni et al., 2020). It was observed that post-transplant cyclophosphamide (PTCy) patients showed healthy MDSC recovery, particularly PMN-MDSCs than standard of care (SOC) recipients, with active T-cell suppressive function (Oshrine et al., 2022). For the first time, it was also found that mitochondrial permeability transition pore (MPTP) opened in the PMN-MDSCs is due to the intense inflammatory environment of GVHD causing mitochondrial damage, oxidative stress, and apoptosis of PMN-MDSCs. By obstructing MPTP opening by cyclosporine A (CsA) (an immunosuppressant), restored the immunosuppressive function and viability of PMN-MDSCs *in vitro* and *in vivo*. Therefore, MPTP blockade by CsA can preserve/active PMN-MDSCs which can improve efficiency bone marrow transplantation. Also, restoration of immunosuppressive function of PMN-MDSCs by enhancing mitochondrial health could serve as a novel therapeutic strategy for aGVHD (Figure 2).

Cyclosporin A blocks mitochondrial permeability transition pore (MPTP) of PMN-MDSCs therefore, inhibiting MDSCs damage in GVHD inflammatory environment (Li et al., 2022). These studies strongly attest that MDSCs could enhance the function of immune suppressive drug in GVHD. Therefore, more studies are required to confirm whether MDSCs regulating GVHD incidence regulates tumor suppression and whether MDSCs can cause tumor recurrence?

## 5 Targeting MDSCs in B-cell malignancies

Although MDSCs have a short lifespan, their continuous recruitment to the sites of inflammation enables a significantly long-lasting affect at that TME site. Also, as the life span of MDSCs in the tissue is short, it is very difficult to reverse its pathological state. Therefore, targeting MDSCs for effective therapies could be achieved by 1. Targeting MDSC recruitment, 2. MDSC depletion and 3. reprograming MDSCs to enhance antitumor immunity.

Strategies to target MDSCs in hematological malignancies have provided some initially promising results. STAT3 and cyclooxygenase 2 (COX2)/PGE2 plays an important role in MDSCs formation, differentiation, and accumulation. Veltman et al. (2010) reported ‘celecoxib’, an inhibitor of COX2, as a potential tool to improve dendritic cell based immunotherapy and can also effectively suppress MDSC function. A retrospective population-based study on DLBCL patients showed a survival benefit to COX-2 inhibitors prior to chemo-immunotherapy treatment (Smyth et al., 2020). A pre-clinical study by De Henau et al. (2016) showed that resistance to immune checkpoint blockade (ICB) is corelated with the infiltration of myeloid cells in various tumors. These findings strongly suggest the inhibition of PI3Kγ (gamma isoforms of phosphoinositide 3-kinase, which are highly expressed in myeloid cells) could be a potential target to overcome immune checkpoint blockade resistance in tumor immune landscape. Furthermore, in HL, it has been reported that RP6530, a PI3Kδ/γ inhibitor decreases the MDSC percentage, and downregulates iNOS which results in tumor regression (Locatelli et al., 2019).

Additionally, miRNAs are also known to affect MDSC function. In a B-cell lymphoma mouse model, mir-30a was upregulated M- and G-MDSCs which further increased the immunosuppressive function of MDSCs by inhibiting the expression of the suppressor of cytokine signaling (SOCS3) gene (Xu et al., 2017c). Li et al. (2020) reported R96A, ‘c-Rel’, which is a member of NF-B family, as a myeloid checkpoint for cancer immunotherapy. A deficiency of c-Rel in MDSCs was reported to inhibit cancer growth in mice which was further confirmed by pharmaceutical inhibition of c-Rel. Furthermore, a combinational therapy of c-Rel and PD1 blockade was more effective in cancer treatment than either strategy alone.

Chimeric antigen receptor (CAR) T-cell therapy, also known as a “living drug”, works on the principle of ‘engineering the patient’s T-cells for the treatment of cancer’. This treatment is routinely used in blood cancers, including lymphomas, some forms of leukemia, and, most recently, multiple myeloma (Neelapu et al., 2017; Schuster et al., 2017; Maude et al., 2018). However, Jain et al. (2021) reported that CAR T-cell failure in large B-cell lymphoma (LBCL) was associated with increased numbers of circulating M-MDSC and tumor associated IFN signaling. Some more clinical studies ongoing are listed in Table 2.

**TABLE 2:** Clinical trials performed worldwide for MDSCs (listed in clinicaltrials.gov).

Study	Single agents targeting MDSCs	Cellular/immune therapy (including TRIKEs)	Combination approaches	Status	Clinical Trials.gov identifier	Key outcomes	Sponsor information
Myeloid-derived suppressor cells (MDSCs) in OSCC patients	Dietary supplement: *ß*-glucan	NA	Beta-glucan administration in oral squamous cell carcinoma patients	Unknown	NCT04387682	β-glucan increasing anti-tumor immunity for OSCCs reduces the recurrence rate or improves survival	National Taiwan University Hospital
Targeting myeloid-derived suppressor cells in recurrent glioblastoma: Phase 0/1 trial of low dose capecitabine + bevacizumab in patients with recurrent glioblastoma	Capecitabine and Bevacizumab	Chemotherapy with immunotherapy	Capecitabine given orally, and the cycle length was 28 days	Active. Not recruiting	NCT02669173	To achieve a 20-fold MDSC reduction in the concentration of circulating MDSCs after treatment with low-dose capecitabine	Case Comprehensive Cancer Center
			Treatment continued until disease progressed	Start Date: 21 January 2016			
			Bevacizumab (10 mg/kg) given every 28 days, until progression	Estimated end Date: January 2023			
IMMUNeOCT study: Octreotide LAR in the induction of immunologic response in patient with neuroendocrine tumors: An interventional pharmacological study	Octreotide Acetate	Chemotherapy	Octreotide Acetate administration every 28 days	Completed	NCT04129255	To observe the impact of OCTREOTIDE LAR on the immune response by studying T-Reg and MDSC and the immunoregulatory cell population in peripheral blood of patients with neuroendocrine tumors G1/G2 treated with OCT LAR.	Hangzhou Sumgen Biotech Co., Ltd
				Start Date: 16 October 2019			
				Estimated end Date 10 September 2020			
Tadalafil to overcome immunosuppression during chemoradiotherapy for IDH-wildtype Grade III-IV Astrocytoma	Tadalafil	Chemotherapy, radiation therapy and adjuvant therapy	Tadalafil given orally once daily for a total of 60 days	Active, not recruiting	NCT04757662	Relative change of MDSCs in peripheral blood	Washington University School of Medicine
			Radiation therapy (RT) to 60 Gy in 30 daily fractions will be administered in this study	Start Date: 17 February 2021			
			And adjuvant TMZ will be initiated for 4–6 weeks after completion of RT (6 cycles at 150–200 mg/m^2 PO per day on Days 1–5 of every 28-day cycle)	Estimated end Date 10 June 2022			
SX-682 treatment in subjects with metastatic melanoma concurrently treated with pembrolizumab	SX-682 in combination with pembrolizumab	Chemotherapy	SX-682 Maximum Tolerated Dose (MTD) during Monotherapy Stage [ Time Frame: Up to 21 Days in 21-day Cycle 1 of Monotherapy Stage. ]	Recruiting	NCT03161431	Biomarker measurement including, tumor MDSC, Tregs and CD69/CD8 T cells, and in the circulation, T- and B-cell subpopulations, neutrophils, the neutrophil-to-lymphocyte ratio (NLR), Tregs, the CD4:CD8 ratio, chemokines, cytokines, and LDH	Syntrix Biosystems, Inc
		SX-682- a selective inhibitor of C-X-C Motif Chemokine Receptor 1 (CXCR1) and C-X-C Motif Chemokine Receptor 2 (CXCR2)		Start Date: 19 May 2017			
		Pembrolizumab- targets the programmed cell death 1 receptor (PD-1)		Estimated end Date 8 February 2023			
Study evaluating the influence of LV5FU2 bevacizumab plus anakinra association on metastatic colorectal cancer (IRAFU)	LV5FU2 Bevacizumab plus anakinra	Chemotherapy and anti-angiogenic therapy		Completed	NCT02090101	1.Median progression-free and overall survival were 5.4 (95% CI, 3.6–6.6) and 14.5 months (95% CI, 9–20.6), respectively	Centre Georges Francois Leclerc
			2-week cycles of bevacizumab + LV5FU2 (folinic acid + 5-FU) + anakinra	Start Date: 18 March 2014		2.Common grade 3 adverse events were neutropenia in 25% of patients, digestive side effects in 21.9 patients, and hypertension in 18.75%	
		IL-1β and *a* inhibitor		Estimated end Date: 9 August 2018			
RTA 408 capsules in patients with melanoma—REVEAL	Combination With Nivolumab/ipilimumab	Chemotherapy Omaveloxolone targets Nrf2 pathway	Omaveloxolone Capsules (2.5 mg/capsule)		NCT02259231	Omaveloxolone was considered up to 150 mg in combination with checkpoint inhibitors	Reata Pharmaceuticals, Inc
			Ipilimumab (3 mg/kg)	Completed			
			Nivolumab (240 mg)	Start Date: October 2014			
			Omaveloxolone Capsules (10 mg/capsule)	Estimated end Date: 24 June 2021			
			Omaveloxolone Capsules (50 mg/capsule)				
Ipilimumab and All-trans retinoic acid combination treatment of advanced melanoma	Ipilimumab (anti-CTLA-4 and PD-1) and VESANOID (all-trans retinoic acid)	Chemotherapy	Ipilimumab- 4 doses of either 3 or 10 mg/kg ipilimumab every 3 weeks	Active, not recruiting	NCT02403778	1. Number of Adverse Events	University of Colorado, Denver
			VESANOID- 150 mg/m2 orally for 3 days	Start Date: 17 December 2015		2. MDSC frequency and suppressive function	
PDE5 Inhibition *Via* tadalafil to enhance anti-tumor mucin 1 (muc1) vaccine efficacy in patients with HNSCC	Tadalafil	Chemotherapy	Tadalafil-Course 1–10–20 mg tablets orally for 19 consecutive days. In Courses 2 to 4- Tablets were administered daily for 14 consecutive days	Completed Start Date: 9 September 2015 Estimated end Date: 15 June 2021	NCT02544880	1.Adverse events and/or treatment limiting-toxicities after receiving protocol therapy. 2. Rate of tumor-specific immune response to protocol therapy	Donald T. Weed, MD, FACS
	Biological: Anti-MUC1 Vaccine						
	Biological: Anti-Influenza Vaccine						
	Other: Tadalafil Placebo						
	Other: Anti-MUC1 Vaccine Placebo						
	Other: Standard of Care Treatment						
	Other: Anti-Influenza Vaccine Placebo						
Tolerability and safety of HF1K16 injection in patients with refractory solid tumors	HF1K16	Chemotherapy	Dose escalation cohort of HF1K16 given QOD at 45 mg/m2, 90 mg/m2, 120 mg/m2, 160 mg/m2	Recruiting Start Date: 24 May 2022	NCT05388487	1.Incidence of Adverse Events	High Field Biopharmaceuticals Corporation
						2. Incidence of dose-limiting toxicities	
						3.Whole blood profiling	
						4. MDSC expression	

## 6 Conclusion

Emerging evidence suggests a significant role for MDSCs in maintaining homeostasis in the immune system. Research studies have explored the immunosuppressive function of MDSCs and identified their pro-tumor characteristics as well as their negative prognostic impact for patients with malignant hematological diseases. In contrast, MDSCs may also have a favorable role in hematological diseases, for example, by preventing GVHD after HSCT (Figure 2). With the clear understanding of the origin, development, and differentiation of MDSCs, the genomic and metabolic mechanisms could be manipulated to optimize the relationship between MDSCs and other cells in the tumor microenvironment and utilize their function to improve patient outcomes. Aside from the role of MDSCs in suppressing the antitumor immune response in the TME, MDSCs also substantially impact the efficacy of immunotherapy, particularly CAR-T cell treatment (Lindo et al., 2021; Tumino et al., 2021). Several studies have developed ways to inhibit or remove MDSCs by modulating their differentiation (Luo et al., 2022), or by developing NK cells expressing chimeric activated receptors that eliminate MDSCs and improve the anti-tumor affects of CAR-T cells (Parihar et al., 2019). Therefore, treating hematological malignancies by targeting MDSCs will be an important future therapeutic direction. However, a critical challenge will be how best to identify and then target specific subsets of MDSC to improve the clinical outcome of patients. Clearly, substantial additional research is needed to achieve this goal.
